# Prospective Clinical and Molecular Evaluation of Potential *Plasmodium ovale curtisi* and *wallikeri* Relapses in a High-transmission Setting

**DOI:** 10.1093/cid/ciz131

**Published:** 2019-04-19

**Authors:** Mirjam Groger, Luzia Veletzky, Albert Lalremruata, Chiara Cattaneo, Johannes Mischlinger, Rella Manego Zoleko, Johanna Kim, Anna Klicpera, Elias L Meyer, Daniel Blessborn, Markus Winterberg, Ayola A Adegnika, Selidji T Agnandji, Peter G Kremsner, Benjamin Mordmüller, Ghyslain Mombo-Ngoma, Hans-Peter Fuehrer, Michael Ramharter

**Affiliations:** 1 Department of Tropical Medicine, Bernhard Nocht Institute for Tropical Medicine and I. Department of Medicine, University Medical Center Hamburg-Eppendorf, Germany; 2 Centre de Recherches Médicales de Lambaréné, Gabon; 3 Institut für Tropenmedizin, Universität Tübingen, Germany; 4 Center for Medical Statistics, Informatics, and Intelligent Systems, Section for Medical Statistics, Medical University of Vienna, Austria; 5 Centre for Tropical Medicine and Global Health, Nuffield Department of Medicine, University of Oxford, United Kingdom; 6 Mahidol-Oxford Tropical Medicine Research Unit, Faculty of Tropical Medicine, Mahidol University, Bangkok, Thailand; 7 German Center for Infection Research (DZIF), partner site Tübingen, Germany; 8 Institute of Parasitology, University of Veterinary Medicine Vienna, Austria; 9 German Center for Infection Research (DZIF), partner site Hamburg-Luebeck-Borstel, Hamburg, Germany

**Keywords:** *Plasmodium ovale*, *Plasmodium ovale curtisi*, *Plasmodium ovale wallikeri*, relapse, CERMEL

## Abstract

**Background:**

*Plasmodium ovale curtisi* and *wallikeri* are perceived as relapsing malarial parasites. Contrary to *Plasmodium vivax*, direct evidence for this hypothesis is scarce. The aim of this prospective study was to characterize the reappearance patterns of ovale parasites.

**Methods:**

*P. ovale* spp. infected patients were treated with artemether-lumefantrine and followed biweekly for up to 1 year for the detection of reappearing parasitemia. Molecular analysis of reappearing isolates was performed to identify homologous isolates by genotyping and to define cases of relapse following predefined criteria.

**Results:**

At inclusion, 26 participants were positive for *P. ovale curtisi* and/or *P. ovale wallikeri*. The median duration of follow-up was 35 weeks. Reappearance of the same *P. ovale* species was observed in 46% of participants; 61% of *P. ovale curtisi* and 19% of *P. ovale wallikeri* infection-free intervals were estimated to end with reappearance by week 32. Based on the predefined criteria, 23% of participants were identified with 1 or 2 relapses, all induced by *P. ovale curtisi*.

**Conclusion:**

These findings are in line with the currently accepted relapse theory inasmuch as the reappearance of *P. ovale curtisi* strains following initial blood clearance was conclusively demonstrated. Interestingly, no relapse of *P. ovale wallikeri* was observed.

## Teaser

The formation of hypnozoites and consequential relapse of tertian malaria is a widely accepted theory that lacks, however, confirmation for *Plasmodium ovale* spp. This study clinically and molecularly evaluates reappearing *P. ovale* spp. parasites to provide new evidence.

According to current understanding, a major difference between tertian malaria and other human pathogen malaria species is the formation of liver dormancies [1]. It is assumed that these dormancies form in the exo-erythrocytic multiplication phase in the liver, which has been experimentally shown for *Plasmodium vivax* but never been directly proven in the human host for *ovale* malaria [2, 3]. *Plasmodium ovale* was first morphologically described by Stephens in 1922 [4, 5]. Over time, different approaches and theories have been established to explain the reappearance of *P. ovale* spp. Suggested places of schizogony and/or dormancy were, for example, monocytes, cells of the reticulo-endothelial system and the epidermis [6, 7]. Another theory suggested 2 tissue phases: a first phase characterized by the development of so-called cryptozoites that released parasites in the blood stream and a second phase, which would only stem from a limited number of malaria parasites, being responsible for relapses [8]. Finally, the exo-erythrocytic cycle was assigned to the liver [9, 10], and Krotoski set forth today’s generally accepted paradigm of liver dormancy [11]. More recent publications propose the possibility of relapse from an origin different than the liver and suggest that all *Plasmodium* parasites pathogenic to humans might be capable of different forms of reappearance [12–14]. Aside from case reports, the relapse phenomenon of *ovale* malaria lacks scientific confirmation [2, 15, 16]. Relapse events of *P. ovale* spp. reported in the literature predominantly rely on microscopic diagnostics and medical history [2]. Molecular analyses to distinguish between *P. ovale curtisi* and *P. ovale wallikeri* are mostly missing to confirm the diagnosis “relapse” [2, 16, 17]. The aim of this study was to investigate the incidence patterns of ovale reappearance focusing on the molecular characterization of potential relapses and considering the difference between the sympatric species *P. ovale wallikeri* and *P. ovale curtisi*.

## METHODS

This study was conducted at the Centre de Recherches Médicales de Lambaréné (CERMEL) in Gabon [18] as part of a clinical trial investigating the efficacy of artemether-lumefantrine (AL) against uncomplicated non-*falciparum* mono infections and mixed *Plasmodium* infections described elsewhere [19]. AL was administered following primary inclusion and microscopically reappearing parasitemia due to the absence of prolonged post-treatment suppressive activity of blood stage malaria parasites. Primaquine was not administered throughout the study. Blood obtained during screening was manually extracted using the QIAamp® DNA Blood Mini Kit (Qiagen, Hilden, Germany) and analyzed at CERMEL using different in-house polymerase chain reaction (PCR) assays specific for *P. ovale wallikeri* and *P. ovale curtisi* to determine eligibility for long-term follow-up. Results were validated at the Institute for Tropical Medicine, Tübingen, applying methods detailed below. In case of ovale positivity, participants were followed up biweekly for up to 1 year. During these visits, malaria signs and symptoms were assessed, and blood smears as well as 20 µl blood spots on filter paper were collected. Plasma was sampled for the determination of the day 7 (D7) lumefantrine concentration to provide evidence of adequate exposure to blood schizonticidal therapy and to rule out recrudescence from blood stage parasites. At screening and unscheduled visits, a physical examination, measurement of vital signs, and venous blood sampling were added. Venous blood was further drawn on days 28 and 42 and preceding AL administration. The duration of follow-up was calculated using the dates of first and last visit.

### Determination of Lumefantrine Plasma Concentration

Drug concentrations were determined using an liquid chromatography-mass spectrometry/mass spectrometry based assay, validated according to US Food and Drug Administration guidelines. In brief, plasma sample preparation was performed by protein precipitation using a Hybrid Solid Phase Extraction-Precipitation 96-wellplate plate (Supelco) and a Freedom EVO liquid handler system (Tecan). Internal standards were used to compensate for recovery and matrix effects. The extracted drugs were separated using a Dionex Ultimate 3000 ultra high performance liquid chromatography system (Thermo Fisher) equipped with a Zorbax SB-CN column (Agilent). An API500 0 triple-quadrupole mass spectrometer and Analyst 1.6.3 software (both ABSciex) were used for drug detection and quantification. The lower limit of quantification was 9.71 ng/mL for lumefantrine and 1.01 ng/mL for desbutyl-lumefantrine. Three replicates of quality control samples at low, middle, and high concentrations were included in the analysis to ensure precision and accuracy. The total coefficient of variation of all quality control samples were <8% during drug quantification of clinical samples.

### Purification of Nucleic Acids and *Plasmodium* spp. Determination

Nucleic acids (NAs) of dried blood spots were extracted using the QIAamp DNA Mini Kit (Qiagen, Hilden, Germany). Purification of NAs in full blood samples was executed as described earlier [19]. The assays were tested and validated in accordance to the MIQE guidelines [20] for their specificity and showed no cross-species amplification. Pipetting and setup of assays was done with the QIAgility (Qiagen, Hilden, Germany) and manually in sterile workstations. Screening for the presence of *Plasmodium* spp. was performed by ultrasensitive Pan-*Plasmodium* reverse transcription quantitative PCR amplifying the small subunit ribosomal RNA gene (18S) [21]. Species differentiation of pan-*Plasmodium* positive samples was performed as reported previously [19].

### Genotype Assessment and Median-joining Networks

Molecular analysis of several gene loci was conducted for *P. ovale* spp. isolate specification. Initially, *P. ovale* spp. specific PCRs within the nuclear SSU rRNA gene were conducted (Primers rOVA1WC/rOVA2WC) [22]. Positive samples were further specified with PCRs specific for *P. ovale wallikeri* (rOVA1v/rOVA2v) [23] and *P. ovale curtisi* (rOVA1/rOVA2) [24]. Furthermore, ovale positive samples following the just described nested PCR (nPCR) were genotyped using PCRs (potra, porbp2) as reported previously [25–27]. All PCR positive products were Sanger sequenced at LGC Genomics (Berlin, Germany). Already published 18S sequences of *P. ovale curtisi* were obtained by performing a Basic Local Alignment Search Tool search in National Center for Biotechnology Information GenBank. GenBank sequences (20 samples) and those of the present study were aligned in Bioedit v.7.0.8 [28] and trimmed to a length of 624bp. Median-Joining networks were calculated with Network v.5.1.0.0 (Fluxus Technology Ltd., Suffolk, England) applying the default settings. Networks were graphically prepared and supplemented by information on geographic origin in Network Publisher v.2.1.1.2 (Fluxus Technology Ltd.), and finalized in Adobe Illustrator CC v.19.0.0 (San José, California, USA).

### Reappearance and Relapse Definition

Reappearance can be caused by reinfection, recrudescence, or relapse. Relapse is defined as renewed asexual parasitemia originating from liver dormancies [12] and needs to be distinguished from recrudescence, which is reappearance that “refers to renewed manifestation of malaria attributed to survival (with or without treatment) of erythrocytic forms” [29]. In this study, reappearance time was the infection-free interval between 2 positive PCR measurements given at least 1 negative PCR measurement in between. In case of several positive measurements before the first negative measurement, the last positive measurement was chosen. Relapse was defined as reappearance of identical *P. ovale* spp. genotypes confirmed in 2 or more genes, following adequate AL treatment with, if available, a D7 lumefantrine plasma level ≥ 280 mg/dL [30] and at least 1 follow-up blood sample with PCR confirmed ovale negativity between the 2 episodes. The wording “confirmed in [n] genes” describes that isolates of [n] genes were identifiable and identical. In case of nonconfirmation sequence analysis failed as parasitemias were too low, isolates were not identical or because genes could stem from coinfecting strains. The term “relapse” therefore describes a potential relapse postulated on the basis of the above-mentioned criteria. The baseline ovale infection is defined as primary infection.

### Statistical Analysis and Data Management

**Figure 1.**
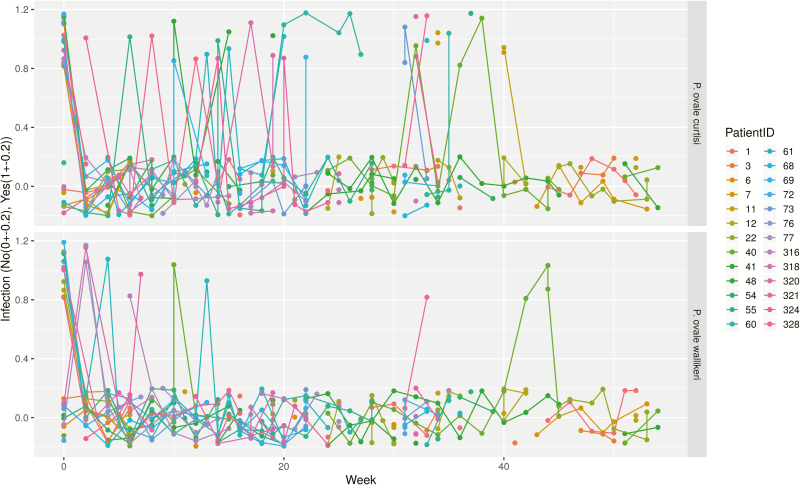
Patient profile/timeline plot depicting reappearance patterns throughout the observational period. The *x*-axis shows the time in weeks, the *y*-axis displays *Plasmodium ovale* spp. infection as categorical variable (yes/no). For better visibility, infection variables are jittered (no: −0.2 to 0.2; yes: 0.8–1.2). Missing values are represented by gaps.

**Figure 2.**
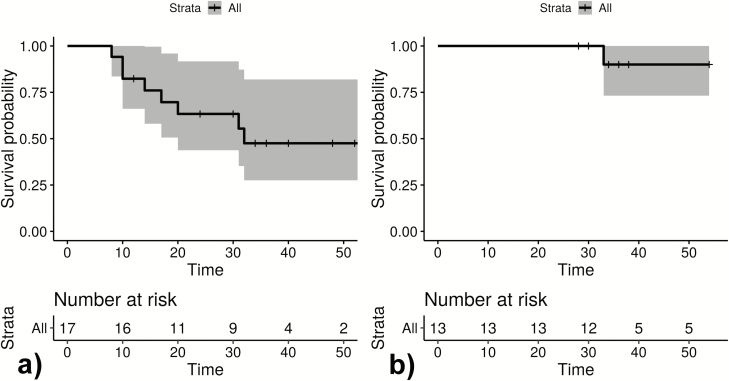
Infection-free intervals until first (quantitative polymerase chain reaction positive) reappearance in weeks of *Plasmodium ovale curtisi* parasites (*A*) and *Plasmodium ovale wallikeri* parasites (*B*). The unit of interest is participants. + Loss to follow-up; gray area: 95% confidence interval.

**Figure 3.**
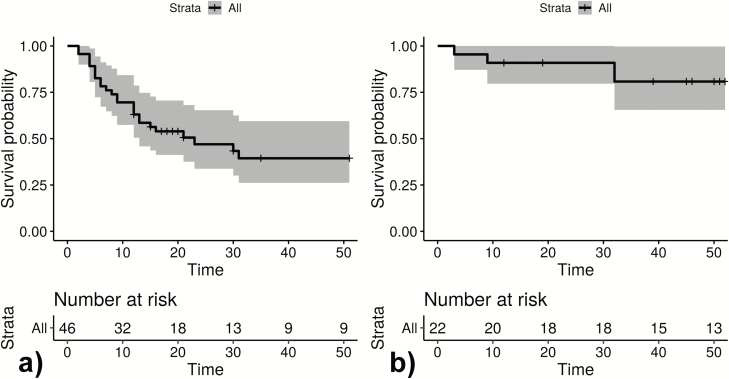
Infection-free intervals until (quantitative polymerase chain reaction positive) reappearance in weeks for all observed reappearances of *Plasmodium ovale curtisi* parasites (*A*) and *Plasmodium ovale wallikeri* parasites (*B*). Following drop out due to reappearance, participants are reincluded in the Kaplan-Meier blots. The unit of interest is infection-free time to reappearance. + Loss to follow-up; gray area: 95% confidence interval.

**Figure 4.**
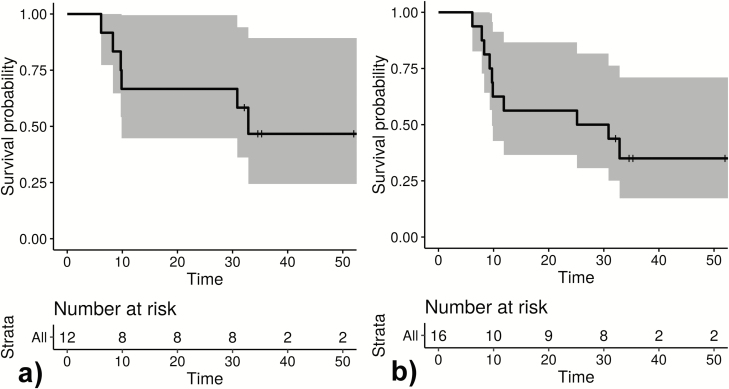
Kaplan-Meier blots show time in weeks to first relapse of *Plasmodium ovale curtisi* measuring participants (*A*) and all observed relapses of *P. ovale curtisi* parasites measuring time to relapse (*B*).

This study was designed to describe relapse patterns of *P. ovale* spp. Since there was no formal hypothesis testing, no formal sample size calculation was performed. For the 26 included participants, a patient profile/timeline plot was drawn to depict reappearance of *P. ovale curtisi* and *P. ovale wallikeri* in detail (Figure 1). Additionally, Kaplan-Meier plots showing infection free times to reappearance were drawn. Therein, missing values were considered to be negative measurements. Infections with other species within the observational period were not considered. Figure 2 shows infection free intervals until first quantitative PCR (qPCR)-based reappearance. In Figure 3, participants were reincluded following drop-out due to qPCR-determined reappearance of the same ovale species allowing for multiple reappearences per participant. The first 4 weeks following any positive microscopic measurement (of any species) were censored. Kaplan-Meier plots were further created to depict infection free times until relapse (Figure 4). Statistical analyses were performed using R 3.4.3 and IBM^®^ SPSS^®^ Statistics 23.

## RESULTS

Between October 2014 and October 2016, 34 patients with PCR corrected *P. ovale* spp. infections were included, and 537 samples were analyzed by ultrasensitive real-time qPCR. Three patients withdrew from the study shortly after inclusion, and 5 were negative for ovale malaria following validation by qPCR. For the remaining 26 participants, the female/male ratio was 1. Mean age was 8.2 years with a range of 2–81 years. The median follow-up period was 35 weeks (IQR: 32–52). Thirteen participants presented with a qPCR diagnosed *P. ovale curtisi* infection at baseline, 9 were positive for *P. ovale wallikeri,* and 4 were positive for both. No major differences in malaria signs and symptoms were observed. Although 24 reappearances of *P. ovale curtisi* were seen within the observational period, 4 were observed for *P. ovale wallikeri* (Figure 3). Eighteen participants had adequate D7 lumefantrine concentrations, 1 was below the lower limit of quantification (REP316), 1 was 265 ng/mL (REP318), and for 6 participants no sample was available.

When comparing the results of qPCR and conventional nPCR, it became evident that 5 qPCR positive *P. ovale wallikeri* coinfections were not detected by conventional nPCR. In return, 1 conventional nPCR and sequencing confirmed *P. ovale curtisi* infection was not detected by qPCR (submicroscopic *P. ovale* spp. infection) and 1 possible mismatch occurred (see Supplementary Table 1: REP61, week 4; qPCR *P. ovale curtisi* positive, nPCR and sequencing *P. ovale* sp. positive locus in the SSU_WC gene, other genes were *P. ovale wallikeri* positive).

### Descriptive Depiction of Recurrent *P. ovale* spp. Infections

Figures 1–3 show infection-free intervals and reappearances of *P. ovale curtisi and P. ovale wallikeri* as defined in the method section. Figure 3 shows that, for example, 61% of *P. ovale curtisi* and 19% of *P. ovale wallikeri* infection-free intervals are estimated to end with reappearance by week 32. For both *ovale* spp., no reappearance of parasites was observed if the infection-free intervals lasted longer than 32 weeks.

### Molecular Evaluation of Relapse Characteristics of *P. ovale* spp. Infections

**Figure 5.**
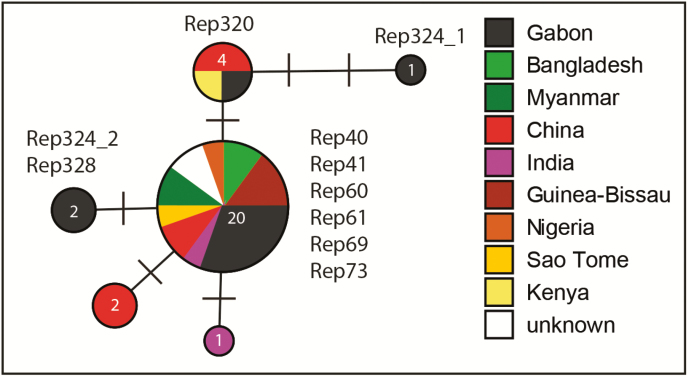
Median Joining network of a 624bp section of the nuclear 18S rDNA gene of *Plasmodium ovale curtisi*, showing the relationships between haplotypes and information on the geographic origin by country. Numbers in the circles indicate the number of individuals sharing the same haplotype, and bars indicate the number of substitutions between haplotypes. Samples are described in detail in Supplementary Table 1 (Rep324_1: Rep324-SCR and D28; Rep 324_2: Rep324-W12).

Twelve patients had ≥1 reappearances of the same *P. ovale* spp. within the observational period. The respective samples (n = 54; 12 baseline samples, 42 follow-up samples) were eligible for genotype assessment. Among the follow-up samples, 6 time points could not be sequenced due to insufficient sample quantity. Two pairs of samples were from the same time point (filter paper from home visit plus blood sample from subsequent unscheduled visit at study site); 1 of each was considered. Two time points were negative when applying the conventional nPCR assay. Among the 32 sequenced samples, 10 (31.3%) fulfilled the predefined criteria for relapse. Four participants had 2 relapses, and 2 participants had 1 relapse. Figure 4 shows infection-free intervals until relapse. Figure 4B, for example, indicates that 53% of *P. ovale curtisi* infection-free intervals of relapses are estimated to end with relapse by week 33. All observed relapses were *P. ovale curtisi* reappearances. The relationships between haplotypes *P. ovale curtisi* and information on the geographic origin by country are outlined in Figure 5. No *P. ovale wallikeri* relapse was observed. Relapse details are outlined in Supplementary Table 1.

## DISCUSSION

This study presents reappearance and relapse patterns of *P. ovale* spp. whose molecular distinction has been mostly left out in previous publications. Without application of sequencing methods, these data indicate a markedly higher overall reappearance of *P. ovale curtisi* as compared to *P. ovale wallikeri*. Focusing entirely on the epidemiological aspects, different scenarios explaining the incidence patterns of the 2 *ovale* species may be hypothesized. The low incidence of observed *P. ovale wallikeri* infections in this study could be explained by a low overall entomologic inoculation rate (EIR) of *P. ovale wallikeri* followed by a low relapse frequency or, alternatively, by a low overall EIR combined with the absence of dormancies. A recent case report of molecularly confirmed *P. ovale wallikeri* relapse in a returning traveler provides evidence that true relapse does occur in that species yet leaves open the origin of the dormancy [16]. Also, studies on returning travelers suggest that *P. ovale wallikeri* does reappear but leave out details about primary and secondary latency [29, 31]. Likewise, the observed *P. ovale curtisi* incidence could be composed of a low EIR followed by frequent relapses, a high EIR accompanied by a low relapse frequency or a balanced occurrence of new infections and relapse. Owing to a lack of EIRs of non-*falciparum Plasmodium* species and other fundamental evidence, these theories remain to be further evaluated. Alternative reasons for reappearance are recrudescence, reinfection with the same parasite strain or so far unexplained reasons.

To the best of our knowledge, this study comprises of the first prospective work-up of *P. ovale curtisi* and *P. ovale wallikeri* reappearances by molecular methods. For *P. vivax*, similar data are already available. A recent study conducted in the Peruvian Amazon showed that 27% of reappearing *P. vivax* infections were relapses with identical genotype. In relation with an amount of 41% potential relapses in this study, it seems that the relapse with identical genotype has a higher significance for the incidence of *P. ovale curtisi* than of *P. vivax* and plays a negligible role for *P. ovale wallikeri*. Further observations of *P. vivax* imply that depending on the geographical location of a strain, typical times to relapse vary between a few months in tropical zones and up to over a year in temperate zones. This geographical pattern also seems to predetermine the periodicity of relapse. Indeed, results of different studies vary considerably [3, 32–34]. It remains open whether this feature likewise applies to *ovale* spp.

Recent publications suggest that relapse might not be a distinctive feature of tertian malaria, but that all human *Plasmodium* species could evoke different types of reappearance [12–14]. Indeed, several reports of *P. falciparum* and *P. malariae* reappearances indicate long-term persistence of the parasites in the human host [35–39]. Despite its increasing importance in malaria elimination settings it remains difficult to determine the contribution of reappearing *Plasmodium* infections to the overall disease burden. These anecdotal reports may only be the tip of the iceberg as reappearances of parasites would not usually stand out in endemic countries due to the higher incidence of new infections.

As shown in Supplementary Table 1, three samples were positive by qPCR but negative by nPCR. This is explainable by a low quantity of parasites in combination with the different target sizes of the assays. Small fragments of degraded DNA are more likely to be detected with real-time primers with a target size of around 200 base pairs, than with primers designed for conventional PCR with target sizes of around 700–1500 base pairs. Furthermore, the resolution of agarose gel is lower than the fluorescence dependent digitized real time curves.

A limitation of this study was the divergence in sensitivity of sample type. DNA concentration was much lower in samples derived from dried blood spots than in samples extracted from whole blood. In 4 events, the negativity of a pre-relapse PCR result was derived from a dried blood spot whereas the relapse identification was based on a whole blood sample (Supplementary Table 1: REP41, week 10; REP55, week 14; REP69, weeks 10 and 22). It is thus possible that the detected parasites had been consistently present but below the limit of detection of the dried blood spot. Another potential limitation is the coinfection of most samples with other *Plasmodium* species and resulting potential—yet to date still unexplained—influences on the reappearance behavior. Only 3 genes (with seven different protocols) were analyzed in this study, and whole genome sequencing of potential relapses is recommended in upcoming studies to prove identity of sampled isolates. The sequencing method used here may have led to overestimation of relapses. Potential relapses heterologous to the primary attack, as previously shown in molecular analyses of *P. vivax* [40], could not be detected with the here-applied methods. As age distribution in the study population was young, the frequency of relapses might be further biased due to lower semi-immunity.

## CONCLUSIONS

This article presents the first prospective work-up to our knowledge of *ovale* relapses derived from a patient cohort using molecular methods. *P. ovale curtisi* occurs more frequently and seems to reappear in shorter time intervals as compared to *P. ovale wallikeri*. The sequencing results suggest the reappearance of the same *P. ovale curtisi* isolates; this is in line with the current relapse theory but at the same time does not permit inference to the origin of the underlying mechanism.

## Supplementary Data

Supplementary materials are available at *Clinical Infectious Diseases* online. Consisting of data provided by the authors to benefit the reader, the posted materials are not copyedited and are the sole responsibility of the authors, so questions or comments should be addressed to the corresponding author.

ciz131_suppl_Supplementary-Material

## Notes

***Acknowledgments.***The authors thank Josef Harl from the Institute of Pathology and Forensic Veterinary Medicine, Department of Pathobiology of the University of Veterinary Medicine Vienna, Austria, for his indispensable help creating the Median-Joining Network. They acknowledge the commitment of the field workers, nurses, and other study personnel for this study. They further thank the study participants and the parents and family members accompanying the young participants for their cooperation.

***Disclaimer.***The funders played no role in study design, data collection and interpretation, or the decision to submit the work for publication.

***Ethical considerations.***The study was conducted according to the ethical principles stated in the Declaration of Helsinki, the applicable guidelines for International Conference on Harmonisation Guideline for Good Clinical Practice, and the applicable laws and regulations of Gabon. The study was approved by the independent ethics committee of the Centre de Recherches Médicales de Lambaréné (Reference number: CEI-CERMEL: 007/2014).

***Financial support.***This work was supported by the Karl Landsteiner Gesellschaft and the Federal Ministry of Science, Research, and Economy of Austria as part of the European and Developing Countries Clinical Trials Partnership (EDCTP)-2 program. This study is part of the EDCTP-2 programme supported by the European Union.

***Potential conflicts of interest.***B. M. reports grants from Deutsches Zentrum für Infektionsforschung, during the conduct of the study. All other authors report no potential conflicts. All authors have submitted the ICMJE Form for Disclosure of Potential Conflicts of Interest. Conflicts that the editors consider relevant to the content of the manuscript have been disclosed.

## References

[1] Mueller I, Galinski MR, Baird JK, et al. Key gaps in the knowledge of *Plasmodium vivax*, a neglected human malaria parasite. Lancet Infect Dis 2009; 9:555–66. 10.1016/S1473-3099(09)70177-X 19695492

[2] Groger M, Fischer HS, Veletzky L, Lalremruata A, Ramharter M A systematic review of the clinical presentation, treatment and relapse characteristics of human *Plasmodium ovale* malaria. Malar J 2017; 16:112. 28284211 10.1186/s12936-017-1759-2 PMC5346189

[3] White NJ Determinants of relapse periodicity in *Plasmodium vivax* malaria. Malar J 2011; 10:297. 21989376 10.1186/1475-2875-10-297 PMC3228849

[4] Stephens J A new malaria parasite of man. Ann Trop Med Parasitol 1922; 16:383–6.

[5] Stephens J, Owen DU Plasmodium ovale. Ann Trop Med Parasitol 1927; 21:293–302.

[6] James SP, Tate P Exo-erythrocytic schizogony in *Plasmodium gallinaceum* Brumpt, 1935. Parasitol 1938; 30:128–38.

[7] Shute PG Protracted incubation periods in indigenous cases of malaria in England. J Trop Med Hyg 1939; 42:201–4.

[8] Davey DG Biology of the malarial parasite in the vertebrate host. Nature 1944; 153:110–1.

[9] Garnham PC Exoerthrocytic schizogony in *Plasmodium kochi* Laveran: a preliminary note. Trans R Soc Trop Med Hyg 1947; 40:719–22. 20243887 10.1016/0035-9203(47)90029-1

[10] Shortt HE, Garnham PC The pre-erythrocytic stage of human malaria, *Plasmodium vivax*. Br Med J 1948; 1:547. 10.1136/bmj.1.4550.547 PMC2090089 18909485

[11] Krotoski WA Discovery of the hypnozoite and a new theory of malarial relapse. Trans R Soc Trop Med Hyg 1985; 79:1–11. 3922096

[12] Markus MB Do hypnozoites cause relapse in malaria? Trends Parasitol 2015; 31:239–45. 25816801 10.1016/j.pt.2015.02.003

[13] Richter J, Franken G, Holtfreter MC, Walter S, Labisch A, Mehlhorn H Clinical implications of a gradual dormancy concept in malaria. Parasitol Res 2016; 115:2139–48. 27079460 10.1007/s00436-016-5043-0

[14] Richter J, Franken G, Mehlhorn H, Labisch A, Häussinger D What is the evidence for the existence of *Plasmodium ovale* hypnozoites? Parasitol Res 2010; 107:1285–90. 20922429 10.1007/s00436-010-2071-z

[15] Fuehrer HP, Habler VE, Fally MA, et al. *Plasmodium ovale* in Bangladesh: genetic diversity and the first known evidence of the sympatric distribution of *Plasmodium ovale curtisi* and *Plasmodium ovale wallikeri* in southern Asia. Int J Parasitol 2012; 42:693–9. 22633951 10.1016/j.ijpara.2012.04.015

[16] Veletzky L, Groger M, Lagler H, et al. Molecular evidence for relapse of an imported *Plasmodium ovale wallikeri* infection. Malar J 2018; 17:78. 29426330 10.1186/s12936-018-2226-4 PMC5807828

[17] Starzengruber P, Fuehrer HP, Swoboda P, et al The first case of *Plasmodium ovale* malaria from Bangladesh. BMJ Case Rep 2010; 2010. pii:bcr0320102865. doi:10.1136/bcr.03.2010.2865 10.1136/bcr.03.2010.2865 PMC3029534 22778371

[18] Ramharter M, Adegnika AA, Agnandji ST, et al. History and perspectives of medical research at the Albert Schweitzer Hospital in Lambaréné, Gabon. Wien Klin Wochenschr 2007; 119:8–12. 17987353 10.1007/s00508-007-0857-5

[19] Groger M, Veletzky L, Lalremruata A, et al Artemether-lumefantrine for the treatment of *Plasmodium malariae*, *Plasmodium ovale*, and mixed *Plasmodium* malaria: a prospective clinical trial assessing species-specific efficacy in Gabon. Antimicrob Agents Chemother 2018; 62: e01758–17. doi:10.1128/AAC.01758-17 10.1128/AAC.01758-17 PMC5826119 29311086

[20] Bustin SA, Benes V, Garson JA, et al. The MIQE guidelines: minimum information for publication of quantitative real-time PCR experiments. Clin Chem 2009; 55:611–22. 19246619 10.1373/clinchem.2008.112797

[21] Mordmüller B, Surat G, Lagler H, et al. Sterile protection against human malaria by chemoattenuated PfSPZ vaccine. Nature 2017; 542:445–9. 28199305 10.1038/nature21060 PMC10906480

[22] Fuehrer HP, Stadler MT, Buczolich K, Bloeschl I, Noedl H Two techniques for simultaneous identification of *Plasmodium ovale curtisi* and *Plasmodium ovale wallikeri* by use of the small-subunit rRNA gene. J Clin Microbiol 2012; 50:4100–2. 23015675 10.1128/JCM.02180-12 PMC3503021

[23] Calderaro A, Piccolo G, Perandin F, et al. Genetic polymorphisms influence *Plasmodium ovale* PCR detection accuracy. J Clin Microbiol 2007; 45:1624–7. 17360843 10.1128/JCM.02316-06 PMC1865880

[24] Snounou G, Viriyakosol S, Zhu XP, et al. High sensitivity of detection of human malaria parasites by the use of nested polymerase chain reaction. Mol Biochem Parasitol 1993; 61:315–20. 8264734 10.1016/0166-6851(93)90077-b

[25] Sutherland CJ, Tanomsing N, Nolder D, et al. Two nonrecombining sympatric forms of the human malaria parasite *Plasmodium ovale* occur globally. J Infect Dis 2010; 201:1544–50. 20380562 10.1086/652240

[26] Oguike MC, Betson M, Burke M, et al. *Plasmodium ovale curtisi* and *Plasmodium ovale wallikeri* circulate simultaneously in African communities. Int J Parasitol 2011; 41:677–83. 21315074 10.1016/j.ijpara.2011.01.004 PMC3084460

[27] Tanomsing N, Imwong M, Sutherland CJ, et al. Genetic marker suitable for identification and genotyping of *Plasmodium ovale curtisi* and *Plasmodium ovale wallikeri*. J Clin Microbiol 2013; 51:4213–6. 24068009 10.1128/JCM.01527-13 PMC3838052

[28] Hall TA BioEdit: a user-friendly biological sequences alignment editor and analysis program for Windows 95/98/NT. Nucleic Acids Symp Ser 1999:95–8.10780396

[29] Rojo-Marcos G, Rubio-Muñoz JM, Angheben A, et al; TropNet Plasmodium ovale investigator group Prospective comparative multi-centre study on imported *Plasmodium ovale wallikeri* and *Plasmodium ovale curtisi* infections. Malar J 2018; 17:399. 30376868 10.1186/s12936-018-2544-6 PMC6208040

[30] Ezzet F, van Vugt M, Nosten F, Looareesuwan S, White NJ Pharmacokinetics and pharmacodynamics of lumefantrine (benflumetol) in acute *falciparum* malaria. Antimicrob Agents Chemother 2000; 44:697–704. 10681341 10.1128/aac.44.3.697-704.2000 PMC89749

[31] Nolder D, Oguike MC, Maxwell-Scott H, et al An observational study of malaria in British travellers: *Plasmodium ovale wallikeri* and *Plasmodium ovale curtisi* differ significantly in the duration of latency. BMJ Open 2013; 3 pii:e002711. doi:10.1136/bmjopen-2013-002711. 10.1136/bmjopen-2013-002711 PMC3657643 23793668

[32] White MT, Karl S, Koepfli C, et al. *Plasmodium vivax* and *Plasmodium falciparum* infection dynamics: re-infections, recrudescences and relapses. Malar J 2018; 17:170. 29665803 10.1186/s12936-018-2318-1 PMC5905131

[33] Chuquiyauri R, Peñataro P, Brouwer KC, et al. Microgeographical differences of *Plasmodium vivax* relapse and re-infection in the Peruvian Amazon. Am J Trop Med Hyg 2013; 89:326–38. 23836566 10.4269/ajtmh.13-0060 PMC3741256

[34] Hankey DD, Jones R Jr, Coatney GR, et al. Korean vivax malaria. I. Natural history and response to chloroquine. Am J Trop Med Hyg 1953; 2:958–69. 13104804

[35] Malvy D, Torrentino-Madamet M, L’Ollivier C, et al *Plasmodium falciparum* recrudescence two years after treatment of an uncomplicated infection without return to an area where malaria is endemic. Antimicrob Agents Chemother 2018; 62 pii:e01892-17. doi:10.1128/AAC.01892-17. 10.1128/AAC.01892-17 PMC5786779 29229635

[36] Al Hammadi A, Mitchell M, Abraham GM, Wang JP Recrudescence of *Plasmodium falciparum* in a primigravida after nearly 3 years of latency. Am J Trop Med Hyg 2017; 96:642–4. 28044045 10.4269/ajtmh.16-0803 PMC5361538

[37] Dierksen J, Al-Ibraheemi A, Wanger A, Chen L *Plasmodium falciparum* recurrence two years after exposure in endemic country: a case report. Ann Clin Lab Sci 2016; 46:433–4. 27466306

[38] Hase R, Uwamino Y, Muranaka K, et al. *Plasmodium malariae* malaria with more than a 4-month incubation period: difficult to distinguish from a relapse of *Plasmodium vivax* malaria. Kansenshogaku Zasshi 2013; 87:446–50. 23984595 10.11150/kansenshogakuzasshi.87.446

[39] Schwartz E, Parise M, Kozarsky P, Cetron M Delayed onset of malaria–implications for chemoprophylaxis in travelers. N Engl J Med 2003; 349:1510–6. 14561793 10.1056/NEJMoa021592

[40] Imwong M, Snounou G, Pukrittayakamee S, et al. Relapses of *Plasmodium vivax* infection usually result from activation of heterologous hypnozoites. J Infect Dis 2007; 195:927–33. 17330781 10.1086/512241

