# Supplementary material for: Prospective Clinical and Molecular Evaluation of Potential Plasmodium ovale curtisi and wallikeri Relapses in a High-transmission Setting
Source: Clin Infect Dis. 2019 Apr 19;69(12):2119–26. doi: 10.1093/cid/ciz131 (PMC6880329; doi:10.1093/cid/ciz131)
Supplement: ciz131_suppl_Supplementary-Material [file ciz131_suppl_supplementary-material.pdf]

Supplementary Table 1.

Patient	Week*	Microscopy	Event treated	Nested real-time qPCR	Ovale specific PCR – positive loci	GenBank IDs	Relapse	Comment<br>Identical isolates[#]
REP11	0	pos (Pf, Pm)	yes	Pf, Pm, Pow	neg	NA	NA	LF D7: 1100 mg/dL
REP11	34	neg	no	Pf, Poc	SSU rRNA (POC)<br>porbp2 (POC)	SSU rRNA: MG847127	no	W40 (SSU, porbp2)
REP11	40	pos (Pf)	yes	Pf, Poc	SSU rRNA (POC)<br>porbp2 (POC)	SSU rRNA: MG847127	no	W34 (SSU, porbp2)
REP40	0	pos (Po)	yes	Pf, Poc	SSU rRNA (POC)<br>porbp2 (POC)<br>potra (POC)	SSU rRNA: MG847128	NA	LF D7: 645 mg/dL<br>W36 (porbp2)<br>W38 (SSU, porbp2)
REP40	32	neg	no	Pf, Poc	neg	NA	no	
REP40	36	neg	no	Poc	porbp2 (POC) weak	NA	no	W0+W38 (porbp2)
REP40	38	neg	no	Poc	SSU rRNA (POC)<br>porbp2 (POC)	SSU rRNA: MG847128	no	W0 (SSU, porbp2[a])<br>W36 (porbp2)
REP40	40	neg	no	Pow	SSU rRNA (POW)<br>porbp2 (POW)	porbp2: MG869596<br>SSU rRNA: MG847121	no	W42+W44 (SSU, porbp2)
REP40	42	neg	no	Pow	SSU rRNA (POW)<br>porbp2 (POW)<br>potra (POW)	porbp2: MG869596<br>SSU rRNA: MG847121	no	W40+W44 (SSU, porbp2)
REP40	44	pos (Pf)	no	Pf, Pow	SSU rRNA (POW)<br>porbp2 (POW)<br>potra (POW)	porbp2: MG869596<br>SSU rRNA: MG847121	no	W40+W42 (SSU, porbp2)
REP41	0	pos (Po)	yes	Pf, Poc	SSU rRNA (POC)<br>porbp2 (POC)<br>potra (POC)	porbp2: MG869599<br>SSU rRNA: MG847129	NA	LF D7: 323 mg/dL<br>W10+W19 (SSU, porbp2)<br>W10 (poctra)
REP41	10	pos (Pf)	yes	Pf, Poc	SSU rRNA (POC)<br>porbp2 (POC)<br>potra (POC)	porbp2: MG869599<br>SSU rRNA: MG847129	yes[3]	LF D7 was adequate~; negative qPCR results between W0 and W10; W0 and W10 show identical strains;<br>W0+W19 (SSU, porbp2)

Patient	Week*	Microscopy	Event treated	Nested real-time qPCR	Ovale specific PCR – positive loci	GenBank IDs	Relapse	Comment Identical isolates[#]
								W0 (poctra)
REP41	15	neg	no	Pf, Poc	porbp2 (POC) weak	NA	no	
REP41	19	pos (Pf, Po)	yes	Pf, Poc	SSU rRNA (POC)<br>porbp2 (POC)	porbp2: MG869599<br>SSU rRNA: MG847129	yes[2]	negative qPCR results between W10 and W19; W0+W10 (SSU, porbp2);
REP55	0	pos (Po)	yes	Pf, Poc, Pow	SSU rRNA (POC, POW)<br>porbp2 (POC)<br>potra (POC, POW)	PoC: porbp2: MG869600; SSU rRNA: MG847130<br>PoW: SSU rRNA: MG847122	NA	LF D7: 1220 mg/dL<br>W6+W14 (POC: SSU, porbp2, potra)
REP55	6	pos (Po)	yes	Pf, Poc	SSU rRNA (POC)<br>porbp2 (POC)<br>potra (POC)	porbp2: MG869600<br>SSU rRNA: MG847130	yes[3]	Adequate LF D7 and neg. PCR between W0 and W6; W0+W14 (POC: SSU, porbp2, potra)
REP55	14	pos (Pf)	yes	Pf, Poc	SSU rRNA (POC)<br>porbp2 (POC)<br>potra (POC)	porbp2: MG869600<br>SSU rRNA: MG847130	yes[3]	neg. PCR between W6 and W14;<br>W0+W6 (POC: SSU, porbp2, potra)
REP60	0	pos (Po)	Inadequate	Pf, Pow	SSU rRNA (POC, POW)<br>porbp2 (POW)<br>potra (POW)	POW: porbp2: MG869597; SSU rRNA: MG847123<br>POC: SSU rRNA: MG847131	NA	POW: W4 (potra, porbp2)<br>POC: W4+22+25+26+35 (SSU)
REP60	4	pos (Po)	yes	Pf, Pow	SSU rRNA (POC, POW)<br>porbp2 (POW)<br>potra (POW)	POW: porbp2: MG869597<br>POC: SSU rRNA: MG847131	no	Inadequate treatment at W0;<br>POW: W0 (potra, porbp2)<br>POC: 0+22+25+26+35 (SSU)
REP60	22	neg	no	Pf, Poc	porbp2 (POC), SSU_rRNA (POC)	SSU rRNA: MG847131	no[1]	POC: 0+4+25+26+35 (SSU)
REP60	25	neg	no	Pf, Poc	porbp2 (POC), SSU_rRNA (POC)	SSU rRNA: MG847131	no	POC: 0+4+22+26+35 (SSU)

Patient	Week*	Microscopy	Event treated	Nested real-time qPCR	Ovale specific PCR – positive loci	GenBank IDs	Relapse	Comment Identical isolates[#]
REP60	26	pos (Pf)	yes	Pf, Poc	SSU rRNA (POC)<br>porbp2 (POC)<br>potra (POC)	porbp2: MG869601<br>SSU rRNA: MG847131	no	Microscopic positivity and treatment of event that was first detected by PCR on W22;<br>POC: 0+4+22+25+35 (SSU)
REP60	35	pos (Pf)	yes	Pf, Poc	porbp2 (POC), SSU_WC (POC)	porbp2: MG869601<br>SSU rRNA: MG847131	no[1]	PCR was negative between W26 and W35<br>POC: 0+4+22+25+26 (SSU)
REP61	0	pos (Pf, Po)	yes	Pf, Pow	SSU rRNA (POW)<br>porbp2 (POW)<br>potra (POW)	porbp2: MG869598<br>SSU rRNA: MG847124	NA	LF D7: 287 mg/dL<br>W4 (SSU, porbp2, potra)
REP61	4	pos (Pm, Po)	yes	Pf, Poc ‡	SSU rRNA (POW)<br>porbp2 (POW)<br>potra (POW)	porbp2: MG869598<br>SSU rRNA: MG847124	no	Time between the events is exactly 28 days – otherwise relapse criteria would have been fulfilled;<br>W0 (SSU, porbp2, potra)
REP61	13	pos (Po)	yes	Pf, Poc, Pow‡	SSU rRNA (POC)<br>porbp2 (POC)<br>potra (POC)	SSU rRNA: MG847132	no	W15+33 (SSU, porbp2, potra)
REP61	15	pos (Po)	yes	Poc	SSU rRNA (POC)<br>porbp2 (POC)<br>potra (POC)	SSU rRNA: MG847132	no	W13+33 (SSU, porbp2, potra)
REP61	33	pos (Pf)	yes	Pf, Poc	SSU rRNA (POC)<br>porbp2 (POC)<br>potra (POC)	SSU rRNA: MG847132	yes[3]	Negative PCR between W15 and W33<br>W13+15 (SSU, porbp2, potra)
REP69	0	pos (Pf, Po)	yes	Pf, Pm, Poc, Pow ‡	SSU rRNA (POC)<br>porbp2 (POC)<br>potra (POC)	SSU rRNA: MG847133	NA	LF D7: 313 mg/dL;<br>W10+W22 (SSU, porbp2, potra)

Patient	Week*	Microscopy	Event treated	Nested real-time qPCR	Ovale specific PCR – positive loci	GenBank IDs	Relapse	Comment Identical isolates#
REP69	10	pos (Pf)	yes	Pf, Poc	SSU rRNA (POC) porbp2 (POC) potra (POC)	SSU rRNA: MG847133	yes[3]	LF D7 was adequate~; negative PCR between W0 and W10 W0+W22 (SSU, porbp2, potra)
REP69	22	pos (Pf)	yes	Pf, Poc	SSU rRNA (POC) porbp2 (POC) potra (POC)	SSU rRNA: MG847133	yes[3]	Negative PCR between W10 and W22 W0+W10 (SSU, porbp2, potra)
REP73	0	pos (Po)	yes	Pf, Pm, Poc, Pow ‡	SSU rRNA (POC) porbp2 (POC) potra (POC)	porbp2: MG869602 SSU rRNA: MG847134	NA	W31 (SSU, porbp2, potra)
REP73	31	pos (Pf)	yes	Poc	SSU rRNA (POC) porbp2 (POC) potra (POC)	porbp2: MG869602 SSU rRNA: MG847134	yes[3]	Negative PCR results between W0 and W31; W0 (SSU, porbp2, potra)
REP316	0	pos (Pf, Po)	yes	Pf, Poc	neg	NA	NA	LF D7: <LLOQ
REP316	6	neg	no	Pf, Pm, Pow	SSU rRNA (POW) porbp2 (POW) potra (POW)	SSU rRNA: MG847125	no	
REP320	0	pos (Po)	yes	Pf, Poc	SSU rRNA (POC) porbp2 (POC) potra (POC)	porbp2: MG869603 SSU rRNA: MG847135	NA	W17+W32 (SSU) W17 (porbp2)
REP320	2	neg	no	Pow	neg		no	Event not treated;
REP320	17	neg	no	Poc	SSU rRNA (POC) porbp2 (POC)	porbp2: MG869603 SSU rRNA: MG847135	no	Previous event microscopically negative and therefore not treated; W0+W32 (SSU) W0 (porbp2)
REP320	32	neg	no	Poc	SSU rRNA (POC)	SSU rRNA: MG847135	no	Previous event microscopically negative and therefore not treated;

Patient	Week*	Microscopy	Event treated	Nested real-time qPCR	Ovale specific PCR – positive loci	GenBank IDs	Relapse	Comment Identical isolates[#]
								W0+W17 (SSU)
REP324	0	neg[†]	no	Pf, Pm, Poc	SSU rRNA (POC)<br>porbp2 (POC)	SSU rRNA: MG847136	NA	W6 (SSU, porbp2)
REP324	4	pos (Pf)	no	Pf, Pow [‡]	SSU rRNA (POC, POW)<br>porbp2 (POW)<br>potra (POW)	PoW: SSU rRNA: MG847126<br>PoC: SSU rRNA: MG847136	no	PoC: W2 (SSU, porbp2)
REP324	12	pos (Pf)	yes	Pf, Poc	SSU rRNA (POC)<br>porbp2 (POC)	SSU rRNA: MG847137	no	PoC: different strain
REP328	0	pos (Pf+Po)	yes	Pf, Pm, Poc, Pow [‡]	SSU rRNA (POC)<br>porbp2 (POC)<br>potra (POC)	SSU rRNA: MG847138	NA	W8+W12+W33 (SSU, porbp2)
REP328	8	neg	no	Pf, Pm, Poc	SSU rRNA (POC)<br>porbp2 (POC)	SSU rRNA: MG847138	yes[2]	Negative PCR between W0 and W8<br>W0+W12+W33 (SSU, porbp2)
REP328	12	neg	yes	Pf, Pm, Poc	SSU rRNA (POC)<br>porbp2 (POC)	SSU rRNA: MG847138	no	No treatment of preceding, microscopically negative event; no negative PCR in between<br>W0+W8+W33 (SSU, porbp2)
REP328	33	neg	no	Poc, Pow [‡]	SSU rRNA (POC)<br>porbp2 (POC)	SSU rRNA: MG847138	yes[2]	Negative PCR between W12 and W33<br>W0+W8+W12 (SSU, porbp2)

Pf *Plasmodium falciparum*; Pm *Plasmodium malariae*; Poc/POC *Plasmodium ovale curtisi*; Pow/POW *Plasmodium ovale wallikeri*; Po *Plasmodium ovale*, PO sp. *Plasmodium ovale* species; ND not done; NA not applicable; W week; LF D7 day 7 lumefantrine plasma concentrations; LLOQ lower limit of quantification; *calculated from date of inclusion and visit date; [1] sameness only confirmed in one gene; [2] confirmed in two genes; [3] confirmed in three genes; [a] quality of this isolate deemed not sufficient to postulate relapse; [#] relapse inclusion criteria based on identical sequences; ~ back-calculated D7 LF plasma concentration using the formula $C(t) = C(0) \times e^{-\lambda t}$ with λ = ln 2 / t_H; [†]preceding visit was microscopically Po positive but PCR analysis of the corresponding sample did not work; [‡]results of qPCR and sequencing differ;
